# Plasma GFAP, NfL, and p-tau181 levels as early biomarkers of dementia in Chinese adults: Shenzhen community cohort study

**DOI:** 10.1007/s40520-025-03001-y

**Published:** 2025-03-26

**Authors:** Chunhua Liang, Xueqin Yan, Jing Tian, Yunzhu Yang, Xiaohua Xiao, Yaohui Huang, Tianfu Wang

**Affiliations:** 1 Department of Geriatrics, Shenzhen Second People’s Hospital, Shenzhen, China; 2 Guangdong Key Laboratory for Biomedical Measurements and Ultrasound Imaging, School of Biomedical Engineering, Medical School, Shenzhen University, Shenzhen, China

**Keywords:** Glial fibrillary acidic protein, Neurofilament light, Phosphorylated-tau181, Clinical value, Early diagnosis, AD disease spectrum

## Abstract

**Background:**

Although blood-based biomarkers can be used to detect early Alzheimer’s disease (AD), population differences affect their clinical value in early diagnosis of the disease spectrum.

**Aims:**

To examine the potential of plasma biomarkers to detect different stages along the AD continuum in a Chinese population.

**Methods:**

We enrolled 113 adults from the Shenzhen community (53 cognitively unimpaired [CU], 45 with mild cognitive impairment [MCI], and 15 with AD). We used the single-molecule array technique to detect the levels of glial fibrillary acidic protein (GFAP), neurofilament light (NfL), and phosphorylated-tau181 (p-tau181), and performed *APOE* genotyping. We assessed the association between plasma biomarkers and cognitive scores, and used receiver operating characteristic curves to measure performance for early AD diagnosis.

**Results:**

The plasma GFAP, NfL, and p-tau181 levels increased significantly in AD and were slightly higher in MCI than in CU (GFAP *p* = 0.811, NfL *p* = 0.909, p-tau181 *p* = 0.696). The plasma GFAP and p-tau181 levels negatively correlated with cognitive scores. Blood markers demonstrated higher performance in identifying AD than CU or MCI. Plasma p-tau181 displayed the highest diagnostic value for AD. Predictions of cognitive impairment were more robust when blood markers were combined with clinical indicators for AD (age, sex, body mass index, years of education, and *APOE ε4* carrier status).

**Discussion:**

The expression of plasma GFAP, NfL, and p-tau181 increased in the AD continuum. Importantly, plasma p-tau181 could identify individuals with AD from the general population, with superior predictive performance when combined with age or sex.

**Conclusions:**

Plasma biomarkers are useful screening indicators for early AD in Chinese adults.

## Introduction

Of the several dementia types, Alzheimer’s disease (AD) is the most common, affecting approximately 60–80% of people with dementia. There are almost 10 million patients with AD in China, and 38.77 million patients with mild cognitive impairment (MCI), with a prevalence rate of 15.5%. AD exerts an enormous burden to persons, families, and the economy, and has become the fifth cause of death in China [1]. Substantial challenges in early diagnosis of AD remain, as the latency period for development of typical clinical symptoms can span 20 years [2]. MCI is an early disease stage between cognitive persistence and dementia and has been considered the important window for early diagnosis and intervention along the AD continuum [3]. The levels of brain amyloid beta (Aβ) and tau proteins can be measured in positron emission tomography (PET) and the cerebrospinal fluid (CSF). However, the high cost of PET examination, radiation exposure, and the invasive prospection of lumbar puncture limit the clinical usability of PET or CSF assessments for identifying AD stage. Evaluation of the levels of blood-based biomarkers (BBB) has been recommended as an early screening tool to help diagnose AD at home and abroad, but regional differences still affect the effectiveness and broad application of the method. Additionally, changes in BBB levels remain poorly understood. Therefore, exploring alterations in BBB levels to increase their diagnostic usability in early AD is of great importance.

Reactive astrocytes, neurological damage, and tau pathology are important mechanisms in AD. Investigations of the levels of relevant biomarkers such as plasma glial fibrillary acidic protein (GFAP), neurofilament light (NfL), and phosphorylated-tau181 (p-tau181) have been widely used to distinguish AD stages [4, 5]. In AD, Aβ amyloid plaques are surrounded by reactive astrocytes, leading to increased expression of GFAP [2, 6]. A previous study confirmed that GFAP is associated with AD progression and cognitive decline [7]. Plasma NfL is a specific molecular protein that constitutes an intermediate filament of axons. It is responsible for axonal transport, maintaining the normal shape of neurons and elasticity of nerve fibers, preventing their breakage, increasing the diameter and structural stability of axons, and improving the conduction speed of axons [8]. Elevated plasma NfL levels suggest neuronal and axonal damage [9]. P-tau181 refers to an ISOFORM of tau that is phosphorylated at that site (residue 181) of the protein, and is a specific indicator of AD, reflecting the deposition of tau protein in the brain. Changes in plasma GFAP, NfL, and p-tau181 levels are consistent with those in CSF and PET tests, which makes the clinical application of these markers possible [9–13]. The levels of these blood markers reflect the pathological process of AD and are closely related to disease progression. The more severe the cognitive impairment, the higher the levels of plasma GFAP, NfL, and p-tau181 [7], and alterations in the plasma GFAP, NfL, and p-tau181 content depends on the stage of subjective cognitive function decline (SCD) and MCI [14].

The main aim of this study was to evaluate the association of plasma GFAP, NfL, and p-tau181 levels with cognitive function and the clinical performance of these markers in the early diagnosis of AD in a real clinical cross-sectional cohort population from the Futian district, Shenzhen city in China.

## Methods

### Participant selection

The researchers recruited a total of 113 participants from January 2023 to December 2023. Participants were sourced from the Department of Geriatric Medicine at Shenzhen Second People’s Hospital as well as several community health centers in Shenzhen, specifically those located in Longwei, Xiamilin, Yuanling, and Fuxin. Participants ranged in age from 45 to 95. Since 1970, a variety of neurocognitive scales have been used to detect cognitive function around the world. At present, the assessment scales for community screening cognitive impairment in China include the Mini-Mental State Examination (MMSE), Montreal Cognitive Assessment (MoCA), the Activities of Daily Living Scale (ADL), and the Clinical Dementia Rating Scale (CDR).The selection of participants in this study followed the process outlined in the “2022 Chinese Expert Consensus on the Assessment of Cognitive Impairment in the Elderly”. Our cohort included 53 individuals with normal cognitive (CU) function, 45 with MCI, and 15 with AD. Participants with CU met the following criteria: MMSE score ≥ 27 points; MoCA score ≥ 26 points; and normal daily life ability. MCI diagnoses fulfilled the 2011 diagnostic criteria issued by the National Institute on Aging (NIA) and the Alzheimer’s Association (AA), which define AD-derived MCI. The patients with AD met the 2011 NIA–AA diagnostic criteria for probable AD dementia and probable AD dementia. Participants were excluded if they met any of the following conditions: cognitive impairment not attributable to AD, Parkinson’s disease, cranial tumors, history of craniocerebral trauma or surgery, significant cerebral hemorrhage or infarction, schizophrenia, bipolar disorder, major depressive disorder, severe organ dysfunction, syphilis, and other specific infections. All participants underwent comprehensive clinical and neurological assessments, and blood samples were collected in a fasting state for the detection of AD blood markers and genetic analysis.

All participants or their legal guardians provided written informed consent, and the study was approved by the Ethics Committee of Shenzhen Second People’s Hospital.

### Measurements of plasma biomarkers using the single-molecule array (Simoa)

K2EDTA plasma samples were obtained by venipuncture. After a 10-min centrifugation at 2,000 rpm within 2 h, the plasma was aliquoted (0.5 mL) in polypropylene tubes and stored at − 80 ℃. The samples were subsequently thawed at room temperature and centrifuged at 10,000 ×*g* for 10 min. The GFAP and NfL levels were quantified using the Simoa Human Neurology 4-Plex E (N4PE) assay. The detection range for GFAP was 0–20,000 pg/mL, while that for NfL was 0–2000 pg/mL. The lower limit of quantitation was 2.89 pg/mL for GFAP and 0.400 pg/mL for NfL. Plasma p-tau181 concentrations were assessed using the Simoa tau-181 V2.1 Kit on the HD-X instrument (Ribose Medical Laboratory), following the manufacturer’s instructions. The limit of quantitation (LoQ) of the p-tau181 kit was established as 2.0 pg/mL (combined coefficient of variability: 15.7%; average recovery: 113.7%; functional LoQ = 8.0 pg/mL), with a limit of detection set at approximately 0.62 pg/mL (range, 0.084–1.211 pg/mL).

### Detection of *APOE* genotypes

Real-time fluorescence quantitative PCR was used to detect *APOE* genotypes in the blood samples. Extract blood genomic DNA according to the instructions of Quanterix Corporation Human Blood Genome Extraction Kit, and the concentration of the extracted DNA was determined using a NanoDrop One microspectrophotometer. According to the instructions of the commercial human *APOE ε2/ε3/ε4* genotyping kit (fluorescent PCR method), detection was carried out on a Hongshi SLAN 96 S real-time fluorescent quantitative PCR instrument.

### Statistical analysis

The SPSS software was used for data analysis, and the GraphPad Prism software for preparing the graph designs. One-way ANOVA was used for the quantitative data that conformed to a normal distribution, and the Kruskal–Wallis test was used to compare data skewness. Qualitative data are expressed as rates (%), and chi-square (χ^2^) tests were used for inter-group comparisons. Spearman correlation analysis was used to evaluate the relationship between blood biomarker levels and cognitive scores. The composite prediction of each index was evaluated using binary logistic regression analysis.The receiver operating characteristic curve (ROC curve) was used to assess the diagnostic power of variables in the regression model, compare the values of the area under the curve (AUC) across different plasma markers, and adjust for clinical factors such as age, sex, body mass index (BMI), years of education, and *APOE ε4* carrier status. Furthermore, the SHAP values of each scale were compared to assess their respective impacts on the model. *p* < 0.05 represents statistically significant differences, and all statistical tests were double-tail tests.

## Results

### Participant characteristics

The age of the 113 participants ranged from 47 to 92 years, and the patients in the AD group were significantly older than those in the MCI or CU group (*p*<0.001). The BMI of participants in the AD group was significantly lower than that in the CU and MCI groups (*p* = 0.026. There were no significant differences in terms of sex, years of education, smoking or drinking behavior, presence of hypertension or diabetes, or *APOE ε4* carrier status among the three groups. Conversely, significant differences in the MMSE and MoCA cognitive scores were noted among the three groups (*p* < 0.001) (Table 1).

**Table 1:** Participant characteristics

	All participants	CU	MCI	AD	*p*
N	113	53	45	15	-
Men, N (%)	48 (42.50%)	21 (39.60%)	20 (44.10%)	7 (46.70%)	0.837
Mean age, years (SD)	66.46 (10.60)	63.60 (9.01)	65.98 (10.42)^c^	78.00 (9.06)^b^	< 0.001
Mean body mass index (SD)	23.63 (3.31)	23.97 (3.07)	23.94 (3.55)^c^	21.52 (2.75)^b^	0.026
Mean education status, years (SD)	9.96 (3.62)	10.19 (3.23)	10.09 (3.50)	8.73 (5.06)	0.562
Smoking behavior, N (%)	28 (24.65%)	14 (26.42%)	8 (17.78%)	6 (40.00%)	0.210
Drinking behavior, N (%)	22 (19.47%)	8 (15.09%)	9 (20.00%)	5 (33.33%)	0.287
Hypertension, N(%)	57 (50.44%)	28 (52.83%)	22 (48.89%)	7 (46.67%)	0.883
Diabetes, n (%)	26 (23.01%)	10 (15.09%)	5 (11.11%)	5 (33.33%)	0.480
*APOE ε4* carrier, N (%)	18 (15.93%)	8 (15.09%)	5 (11.11%)	5 (33.33%)	0.122
Mean MMSE score (SD)	25.64 (6.05)	29.04 (0.10)^a^	25.67 (3.47)^c^	13.53 (7.14)^b^	< 0.001
Mean MoCA score (SD)	22.56 (6.84)	27.47 (1.73)^a^	20.93 (4.13)^c^	10.07 (6.48)^b^	< 0.001
Mean GFAP content, pg/mL (SD)	175.04 (107.11)	157.80 (75.53)	162.72 (91.95)^c^	272.09 (179.19)^b^	0.022
Mean NfL content, pg/mL (SD)	30.86 (23.09)	28.53 (23.15)	29.06 (20.80)^c^	44.46 (26.16)^b^	0.023
Mean p-tau181 content, pg/mL (SD)	2.73 (1.96)	2.39 (1.24)	2.54 (1.95)^c^	4.49 (3.01)^b^	< 0.001

Qualitative data are presented as N (%). Quantitative data are presented as the mean (standard deviation [SD]). *P* values are derived from chi-square and Kruskal–Wallis tests. CU, MCI, AD: normal cognitive function, mild cognitive impairment, and Alzheimer’s disease, respectively; MMSE, Mini-Mental State Examination; MoCA, Montreal Cognitive Assessment; GFAP, glial fibrillary acidic protein; NfL, neurofilament light; a = CU vs. MCI (*p*<0.05), b = CU vs. AD (*p*<0.05), c = MCI vs. AD (*p*<0.05)

### Expression of AD plasma biomarkers in a Chinese population

**Fig. 1:**
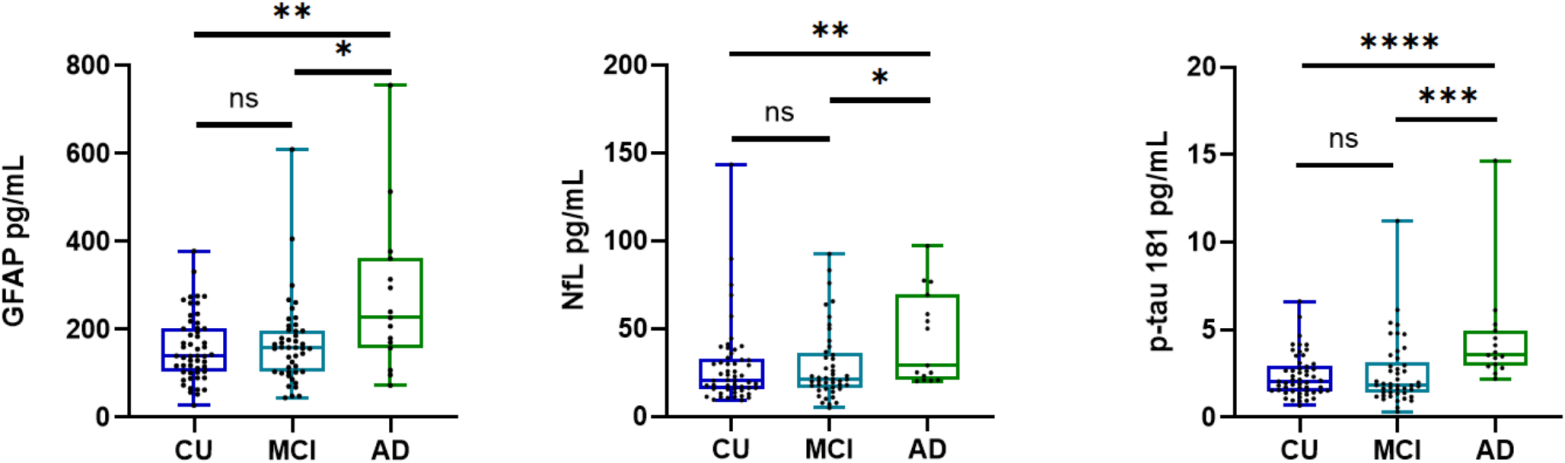
Comparison of the differences in the levels of blood markers between groups. The line segments within each boxplot represent the median, the bottom and top of the box represent 1 SD from the mean, and the whiskers represent the minimum and maximum values. ns indicates a *p* value > 0.05; * indicates a *p* value < 0.05; ** indicates a *p* value < 0.01; *** indicates a *p* value < 0.001; and **** indicates a *p* value < 0.0001

The level of plasma GFAP in the AD group was significantly higher than that in the MCI (*p* < 0.05) and CU groups (*p* < 0.01), but there was no significant difference between the CU and MCI groups (*p* = 0.811). Similarly, the level of plasma NfL in the AD group was significantly higher than that in the MCI (*p* < 0.05) and CU groups (*p* < 0.01), while the difference between the CU and MCI groups was not significant (*p* = 0.909). The level of plasma p-tau181 in the AD group was significantly higher than that in the MCI and CU groups, with the difference between the AD and CU groups being significant (*p* < 0.0001). The level of plasma p-tau181 in the MCI group was slightly higher than that in the CU group, but the difference was not significant (*p* = 0.696)(Table 1; Fig. 1).

### Correlation between plasma GFAP, NfL, and p-tau181 levels and cognitive score

**Fig. 2:**
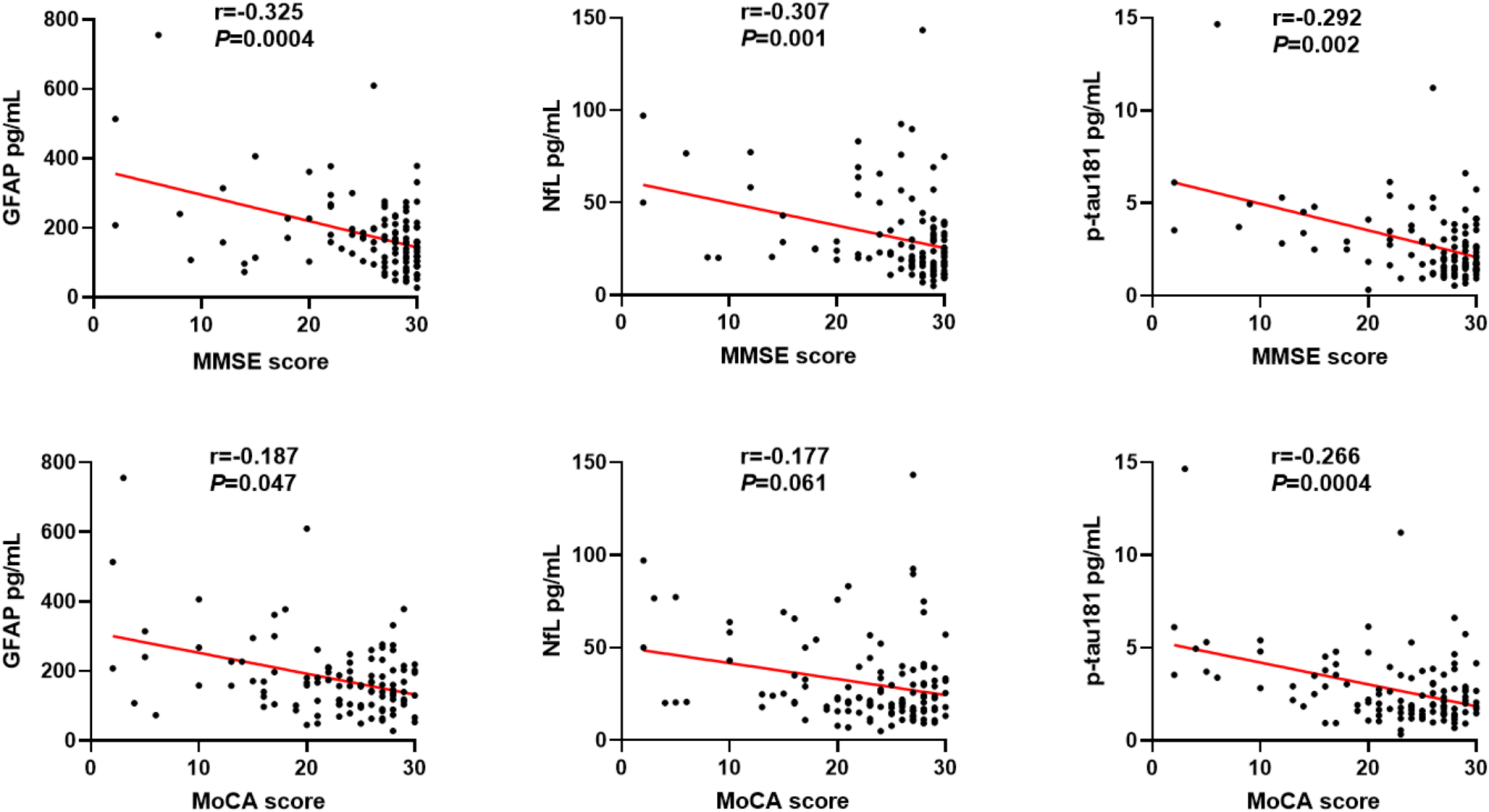
Correlation of plasma GFAP, NfL, and p-tau181 levels with the MMSE and MoCA scores. r indicates the correlation coefficient in Spearman correlation analysis

We observed that the plasma GFAP and p-tau181 levels negatively correlated with MMSE and MoCA scores to a certain extent, with the plasma GFAP level showing the strongest correlation with the MMSE score (*r*= -0.325, *p* = 0.0004). In contrast, the plasma p-tau181 level showed the strongest correlation with the MoCA score (*r*= -0.266, *p* = 0.0004). The plasma NfL content negatively correlated with the MMSE score (*r*= -0.307, *p* = 0.001), whereas no significant association was observed with the MoCA score. (Fig. 2).

### Diagnostic value of plasma GFAP, NfL, and p-tau181 levels

**Fig. 3:**
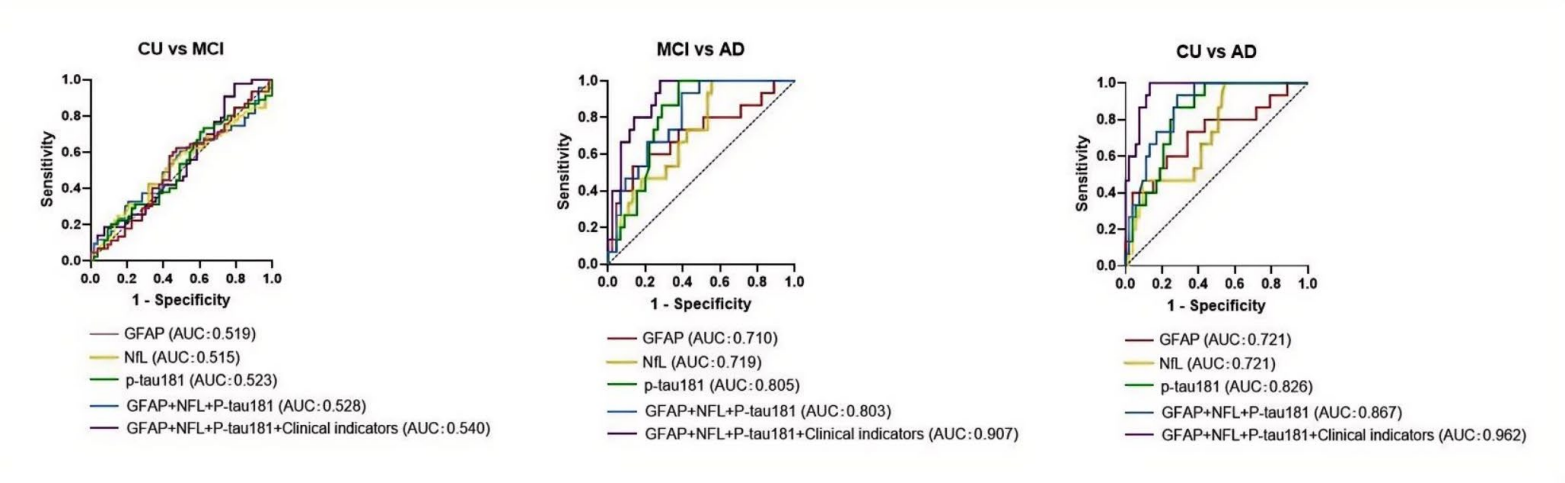
Diagnostic performance in AD of blood biomarkers alone or in combination with other variables. The red, yellow, and green lines represent the performance of plasma GFAP, NfL, and p-tau 181, respectively; the blue line represents the performance of the three plasma biomarkers, whereas the purple line represents the performance of the three markers combined with clinical indicators (age, sex, body mass index, education status, and *APOE ε4* carrier status)

**Fig. 4:**
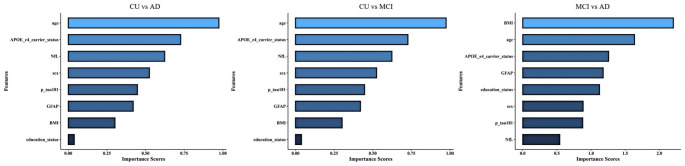
Create variable Importance plot.The length of each bar in the plot is indicative of its significance to the model’s predictions; the longer the bar, the greater its importance

Binary Logistic regression was used to calculate the new variables of the index association, and the latter was analyzed to obtain the AUC. The AUC values for the plasma biomarkers and AD clinical factors (age, sex, BMI, years of education, and *APOE ε4* carrier status) were compared across participants with different cognitive levels to measure the diagnostic value of these parameters in the AD continuum (Fig. 3). And evaluate the importance of each variable in the model through the SHAP value(Fig. 4).

The comparison between CU and MCI produced AUC values for plasma GFAP, NfL, and p-tau 181 of 0.519, 0.515, and 0.523, respectively. The combination of the GFAP, NfL, and p-tau 181 yielded a higher AUC value (0.528) than that for any single plasma biomarker, and after adding clinical indicators of AD, the AUC value for the three proteins was estimated at 0.54. Age exerted the most significant influence on the model.

For MCI vs. AD, the AUC value for plasma p-tau 181 (0.805) was superior to that for GFAP (0.710) or NfL (0.719). After combining the three plasma proteins with clinical indicators of AD, the AUC value increased to 0.907, indicating a high diagnostic value along the AD continuum. BMI exerted the most significant influence on the model.

For CU vs. AD, the AUC value for both plasma GFAP and NfL was 0.721, while that for plasma p-tau 181 was higher (0.826). The AUC value reflecting the synergism of the three proteins (0.867) was superior to that of any single plasma biomarker. Combined with AD clinical indicators such as age, sex, body mass index, education status, and APOE ε4 carrier status, the predicted AUC value was 0.962. Age exerted the most significant influence on the model.

## Discussion

The main goal of this study was to explore alterations in the plasma GFAP, NfL, and p-tau181 levels associated with cognitive impairment in a community-based adult cohort in Shenzhen City, China. The results revealed a trend for gradually increasing expression of plasma GFAP, NfL, and p-tau181 from normal cognitive function to MCI and AD, especially in the AD phase, which was associated with a significantly upregulated expression of these plasma biomarkers. The observed upregulation is consistent with the conclusions of previous research [14]. Furthermore, we found that plasma GFAP, NfL, and p-tau181 levels could distinguish between dementia and non-dementia but not between CU and MCI. In terms of a single plasma biomarker, p-tau181 levels could most strongly identify AD stage. These findings suggest that blood biomarkers can be used as convenient predictors of the AD continuum, providing important evidence for clinical performance in AD dementia.

Previous research has demonstrated that within the AD continuum, the plasma levels of GFAP, NfL, and p-tau181 exhibit a gradual increase, whereas the plasma Aβ1–42/Aβ1–40 ratio displays a progressive decline [14, 15]. In this study, the expression of plasma GFAP, NfL, and p-tau181 in patients with AD was significantly higher than those in the CU or MCI groups, with remarkable differences in pairwise comparisons (CU vs. AD, *p* < 0.0001; MCI vs. AD, *p* < 0.001). Although the levels of plasma GFAP, NfL, and p-tau181 in the MCI group were slightly higher than those in CU, the difference was not significant. Similarly, Ingannato et al. reported that the changes in plasma GFAP and p-tau181 levels between patients with MCI and SCD were not notable [14]. Analysis across the disease spectrum shows that plasma GFAP and p-tau181 levels are remarkably elevated during the AD phase. In this study, the plasma levels of Aβ1–42 and Aβ1–40 were measured using Somia technology to estimate the ratio of Aβ1–42 to Aβ1–40, but no significant differences were observed among the CU, MCI, and AD groups, consequently, the data pertaining to A-beta amyloid is not presented in the table. This further indicates that the plasma levels of GFAP, NfL, and p-tau181 demonstrate superior performance compared to plasma Aβ1–42 and Aβ1–40 levels and the Aβ1–42/Aβ1–40 ratio in the context of cognitive impairment.

Sex and age are important risk factors for AD, with women being more likely to present with the disease than men, and prevalence in women being almost twice that in men [16]. The incidence of AD gradually increases with increasing age, and is 6% in the population at 60 years old but can be as high as 35% in those aged 85 years and older. Both overweight and underweight can increase the risk of dementia [17]. This study revealed that the BMI of participants in the AD group was significantly lower than that in the CU and MCI groups (*p* = 0.026). Individuals with lower educational attainment are at a heightened risk for dementia, too. Although no statistically significant difference was found regarding the years of education among the three groups, our data showed that the AD group received fewer years of education than the MCI group, which, in turn, received fewer years of education than the CU group. It is well established that the major risk factors for developing AD are age and the status of the *APOE ε4* allele [2, 18]. AD risk is determined by genetic factors at a level of 60–80%; the *APOE ε4* allele explains an essential genetic variation in AD and its presence increases the risk of AD by 3–4 times [2, 19]. In this study, the prevalence of *APOE ε4* in the AD group was significantly higher than that observed in the CU and MCI groups; however, prevalence in the MCI group was lower than that in the CU group, which may be related to the insufficient sample size in the study. Future research should aim to incorporate a larger sample size to further elucidate the relationship between APOE ε4 and cognitive function as well as BBB levels.

Interestingly, our correlation analysis of blood biomarkers and cognitive scores revealed that plasma GFAP and p-tau181 levels negatively correlated with the MMSE and MoCA scores to a certain extent, while no significant correlation between plasma NfL level and MoCA score was noted; these findings indicate a closer relationship between the former markers and cognitive function. GFAP levels represent an astrocytic reaction in the process of AD and are elevated in the brains of patients with AD. A systematic review and meta-analysis reported that plasma GFAP levels distinguished between patients with AD and individuals with cognitively normal function, and were strongly associated with brain Aβ pathology; these observations led to speculations for GFAP as a potential blood biomarker for AD [10]. Plasma p-tau181 expression is closely associated with the extensive aggregation of amyloid and tau proteins. The more severe the cognitive impairment, the higher the level of plasma p-tau181 levels, reflecting one of the important diagnostic and screening indicators of AD. In terms of diagnostic performance in AD, plasma p-tau181 showed a superior clinical value (AUC value > 0.8) compared to plasma GFAP and NfL. Bayoumy et al. described that plasma p-tau181 levels could discriminate AD from the control group and this measure has high diagnostic accuracy for AD, with an AUC value of 0.936–0.995 [20]. Moreover, a remarkable finding in this investigation was that the combined capacity of plasma GFAP, NfL, and p-tau181 to predict AD was stronger than individual contributions. Among clinical indicators, age and BMI were found to have a greater impact on cognition. When clinical indicators of AD such as age, sex, BMI, years of education, and *APOE ε4* carrier status were added, the AUC value for predicting AD stage increased to a certain extent, showing a stronger predictive value for the AD spectrum (AUC > 0.9).

Our findings elucidated that plasma GFAP, NfL, and p-tau181 levels are effective diagnostic and predictive biomarkers for the AD continuum. Despite these observations, this study has some limitations. First, the sample size was small, especially of patients with AD. Second, cognitive status and the levels of plasma biomarkers in the participants were measured at a single point in time without further follow-up or assessment of dynamic changes. Lastly, the content of related proteins in PET–computed tomography and CSF was not included, and comparisons between disease biomarkers in the blood and brain could not be performed.

## Conclusion

We described that as cognitive function deteriorates, the expression of plasma GFAP, NfL, and p-tau181 gradually increases. Blood biomarkers can effectively distinguish between dementia and non-dementia states in a Shenzhen community cohort study, with plasma p-tau181 content showing a stronger capacity to predict the clinical spectrum of AD. It is important that any comprehensive analyses of blood biomarkers to diagnose AD consider age, genotype, and other associated factors. Taken together, our findings reveal that plasma GFAP, NfL, and p-tau181 levels are suitable as screening biomarkers for early cognitive impairment in Chinese adults.

## Data Availability

No datasets were generated or analysed during the current study.
